# Structural insights into how vacuolar sorting receptor recognizes the C‐terminal sorting determinant of a vicilin‐like seed storage protein

**DOI:** 10.1111/febs.70245

**Published:** 2025-09-02

**Authors:** Shu Nga Lui, Hsi‐En Tsao, Anthony Hiu‐Fung Lo, Liwen Jiang, Kam‐Bo Wong

**Affiliations:** ^1^ Centre for Protein Science and Crystallography, State Key Laboratory of Agrobiotechnology, School of Life Sciences The Chinese University of Hong Kong Hong Kong China; ^2^ Centre for Cell and Developmental Biology, State Key Laboratory of Agrobiotechnology, School of Life Sciences The Chinese University of Hong Kong Hong Kong China

**Keywords:** crystal structure, protein–protein interaction, receptor‐mediated trafficking, vacuolar sorting

## Abstract

During seed development, vacuolar sorting receptors (VSRs) recognize a sequence‐specific vacuolar sorting determinant located at the C terminus (ctVSD) of storage proteins, thereby sorting them into protein storage vacuoles. The protease‐associated (PA) domain of VSRs is responsible for interacting with the ctVSD of cargo proteins. Here, we report the crystal structure of the PA domain of *Arabidopsis* vacuolar‐sorting receptor 1 (VSR1) in complex with the C‐terminal pentapeptide (_507_SDRFV_511_) of vicilin‐like seed storage protein 22 (VL22). Structural comparison with the apo form of VSR1 reveals conformational changes in four switch regions in the PA domain. VL22 binds to a cradle of VSR1 formed by residues in the cargo‐binding loop, the switch I and III regions. The C‐terminal carboxyl group of VL22 is recognized by forming salt bridges with the invariant Arg95 of VSR1. Compared with the structure of VSR1‐PA in complex with the ctVSD of cruciferin 1, VL22 makes extra hydrophobic interactions with the cargo‐binding loop and hydrogen bonds with switch I residues in VSR1. Tagging the C‐terminal sequence of VL22, but not VL22‐R509P, VL22‐V511P, VL22‐R509P‐V511P nor vicilin‐like seed storage protein 43 (VL43), redirected secretory red fluorescent protein (spRFP) to the vacuoles in *Arabidopsis* protoplasts. Scanning mutagenesis identified an E519S substitution converting the C‐terminal sequence of VL43 to a sorting determinant that can redirect spRFP to the vacuoles, suggesting that charge–charge repulsion prevents the receptor–cargo interactions between VL43 and VSR1. The recognition of ctVSD by VSRs is likely promiscuous, resulting from the additive effect of individual preference of residues in the ctVSD.

## Graphical abstract

Soluble cargo proteins are sorted to the vacuoles via a receptor‐mediated mechanism. Combining crystallographic and mutagenesis studies, we showed how the C‐terminal sorting determinant (ctVSD) of vicilin‐like seed storage protein VL22 is recognized by the protease‐associated domain of *Arabidopsis* vacuolar‐sorting receptor 1. The structural insights can be generalized to understand how seed storage proteins are sorted to the vacuoles. Figure was made using the program pymol.

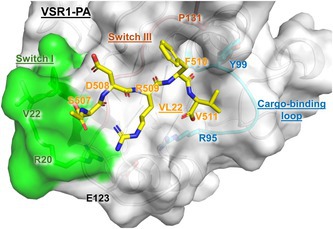

**Abbreviations**
APBS: Adaptive Poisson‐Boltzmann Solver
CHAPS: 3‐[(3‐cholamidopropyl)dimethylammonio]‐1‐propanesulfonate
CRU1: cruciferin 1
ctVSD: C‐terminal vacuolar sorting determinant
EDTA: ethylenediaminetetraacetic acid
MES: 2‐Morpholinoethanesulfonic acid
NHS: N‐Hydroxysuccinimide
NT: N‐terminal
PA: protease associated
PDB: Protein Data Bank
PEG: polyethylene glycol
RMR: receptor‐homology‐transmembrane‐RING‐H2 proteins
r.m.s.d.: root mean square deviation
SDS/PAGE: sodium dodecyl sulfate‐polyacrylamide gel electrophoresis
spRFP: secretory red fluorescent protein
ssVSD: sequence‐specific vacuolar sorting determinant
VL22: vicilin‐like seed storage protein 22
VL43: vicilin‐like seed storage protein 43
VSR: vacuolar sorting receptor

## Introduction

In plant cells, there are two types of vacuoles, namely lytic vacuole and protein storage vacuole [1, 2, 3, 4]. Lytic vacuole is ubiquitous in plant cells. It contains hydrolytic enzymes that are responsible for intracellular digestion, and its function is analogous to lysosomes in animal cells [5, 6]. Protein storage vacuoles, on the other hand, are exclusively found in seeds. During seed development, storage proteins are deposited in the protein storage vacuole to provide nutrients during seed germination [3, 7]. Soluble cargo proteins are sorted to the vacuoles via a receptor‐mediated mechanism [8]. There are two types of sorting receptors in plants: vacuolar‐sorting receptors (VSR) and receptor‐homology‐transmembrane‐RING‐H2 proteins (RMR) [9, 10, 11]. Both VSR and RMR are type I transmembrane proteins containing an N‐terminal lumenal region responsible for cargo binding, a single transmembrane domain, and a C‐terminal region responsible for targeting and subcellular localization [10, 12, 13, 14, 15, 16, 17, 18]. Sorting receptors recognize a sequence‐specific information on the cargo protein known as vacuolar sorting determinant (VSD) and sort them to the vacuoles. Vacuolar sorting in plant cells is dependent on specific receptor–cargo interaction and is different from lysosomal sorting in animal cells that relies on the posttranslational modification of mannose‐6‐phosphate on the cargo proteins [19].

There are two types of VSD: sequence‐specific VSD (ssVSD) that are commonly found in acidic hydrolases and C‐terminal VSD (ctVSD) often found in seed storage proteins [20, 21, 22]. ssVSD contains a “NPIR” motif with the consensus sequence of (N/L)‐(P/I/L)‐(I/P)‐(R/N/S) that is recognized by VSR but not by RMR [23]. How VSR recognizes ssVSD is not fully understood. The N‐terminal lumenal region of VSR contains a protease‐associated (PA) domain, a thioredoxin (TRX) domain, and three epidermal‐growth‐factor (EGF) repeats [9, 24, 25]. The TRX domain was formerly called the Central domain, which was found to be structurally homologous to thioredoxin by crystallographic analysis [26]. We have previously determined the crystal structure of the ssVSD of proaleurain in complex with the PA domain of *Arabidopsis thaliana* vacuolar‐sorting receptor 1 (VSR1‐PA) and showed that the PA domain interacts with the residues preceding the NPIR motif [16]. Both TRX and EGF domains should contribute to cargo binding and release [12, 16, 27], but the mechanism remains poorly understood.

ctVSD is recognized by the PA domain that is found in both VSR and RMR [9, 13, 18, 28, 29, 30]. ctVSD is often found in storage proteins that are targeted to the protein storage vacuoles during seed development [23, 31]. Knocking out *vsr1* and *vsr3*/*vsr4* resulted in mis‐sorting of seed storage protein 12S globulins in *Arabidopsis* seeds [29, 32]. A unique feature of ctVSD is its location at the C terminus. Adding a glycine or proline residue to the C terminus of ctVSD could abolish the receptor–cargo interaction and result in mis‐sorting of the cargo proteins [18, 33, 34]. Although no consensus sequence has been identified, ctVSD typically is rich in hydrophobic residues at the C terminus. For example, the ctVSD of phaseolin, a storage protein of common bean, contains the sequence of AFVY. Removal of these C‐terminal residues led to mis‐sorting of phaseolin to the extracellular space [35]. As an interesting analogy in mammalian cells, the C‐terminal residues of the sorting nexin SNX17 are recognized by the PDZ and LIM domain proteins and the Commander complex to mediate endosomal recycling of transmembrane cargo proteins [36, 37].

We have previously determined the crystal structure of VSR1‐PA in complex with the ctVSD of a 12S globulin cruciferin 1 (_468_RVAAA_472_) [18]. We showed that the C‐terminal carboxyl group of the ctVSD is recognized by an invariant Arg95 residue of the cargo‐binding loop of VSR1, and backbone hydrogen bonds between ctVSD and VSR1 are essential to receptor–cargo interactions. In this study, additional structural insights into how VSR recognizes the ctVSD were obtained from the crystal structure of VSR1‐PA in complex with the ctVSD (_507_SDRFV_511_) of vicilin‐like seed storage protein 22 (VL22). VL22 makes extra interactions with VSR1, inducing it to undergo additional conformational changes. We demonstrate that an E519S substitution can convert the C‐terminal sequence of vicilin‐like seed storage protein 43 (VL43) to a ctVSD recognized by VSR for vacuolar sorting, suggesting that charge–charge repulsion is unfavorable to receptor–cargo interaction. Taken together, our work provides a better understanding of how the PA domain of VSR recognizes the ctVSD.

## Results

### Structure determination of VL22/VSR1‐PA

**Fig. 1:**
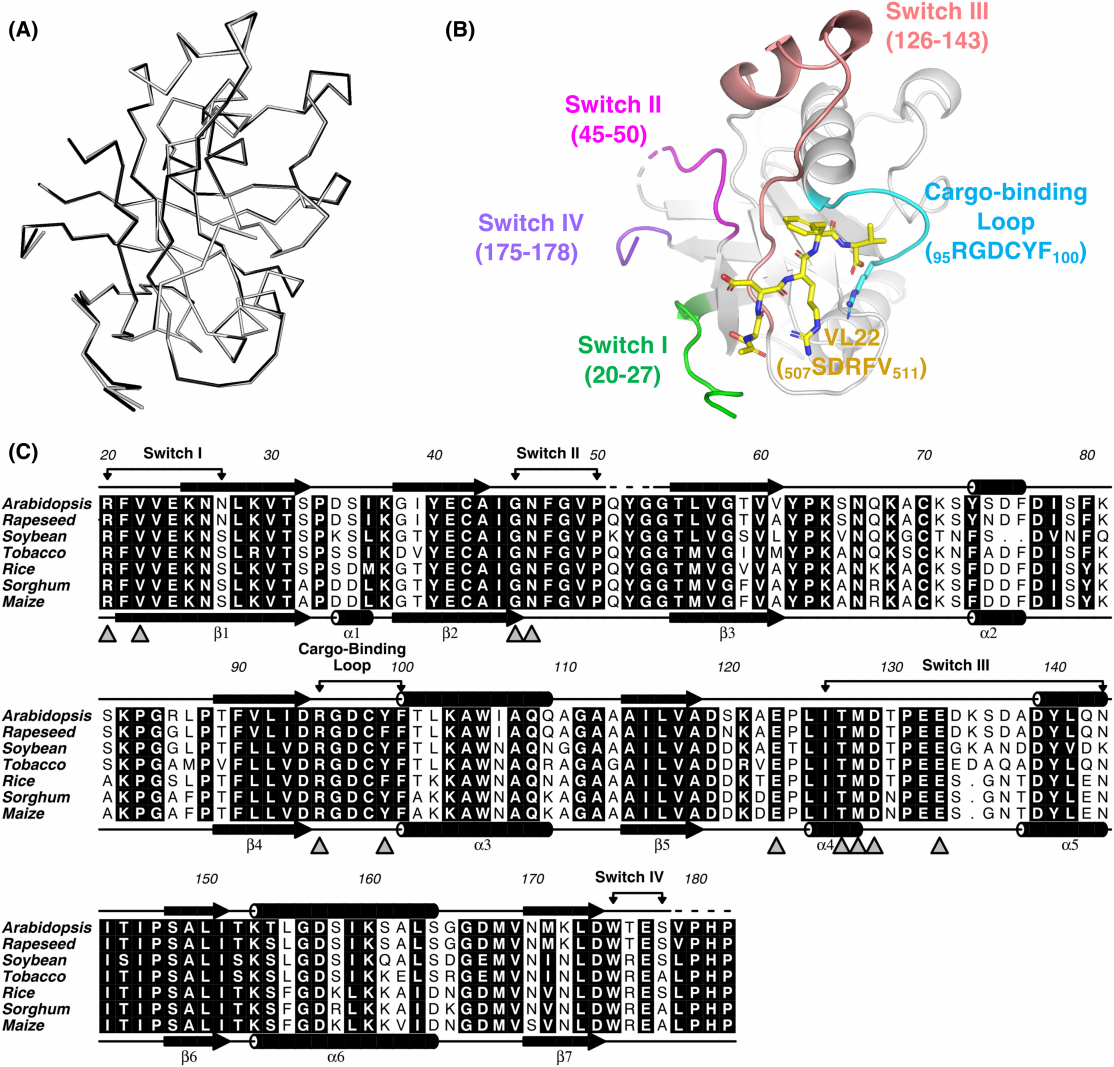
Crystal structure of *Arabidopsis* VSR1‐PA in complex with the C‐terminal pentapeptide of VL22. (A) Structures of vacuolar‐sorting receptor 1 protease‐associated domain (VSR1‐PA) in complex with the C‐terminal pentapeptide (acetyl‐SDRFV) of vicilin‐like seed storage protein 22 (VL22) were determined by co‐crystallization at pH 5.8 (PDB: 9L4O; black) and pH 7.0 (PDB: 9L4P; gray) and displayed as Cα trace. The two structures are essentially identical, with a Cα r.m.s.d. value of 0.167 Å. (B) Cartoon representation of the crystal structure of VL22/VSR1‐PA. Switch I‐IV, the cargo‐binding loop, and VL22 are color coded in green, magenta, salmon, purple, cyan, and yellow, respectively. (C) Sequences of VSR1‐PA of *Arabidopsis thaliana* (NCBI ID; NP_190853.1), rapeseed (*Brassica napus*) (XP_013749989.2), soybean (*Glycine max*) (XP_003552313.1), tobacco (*Nicotiana tabacum*) (XP_016432514.1), rice (*Oryza sativa japonica*) (XP_015616429.2), sorghum (*Sorghum bicolor*) (XP_002450144.1), and maize (*Zea mays*) (XP_008669942.1) were aligned using the program MUSCLE. Conserved residues are in black shading, and those involved in cargo‐binding and conformational changes are indicated by triangles. Secondary structure elements of apo‐ and VL22‐bound VSR1‐PA are shown below and above the alignment, respectively. Disordered residues are indicated by dash lines. Residues are numbered according to the *Arabidopsis* sequence. (A, B) were generated using the program pymol (www.pymol.org).

We previously reported that VSR1 interacts with the ctVSD of a vicilin‐like seed storage protein VL22, and sorts cargo protein containing the ctVSD of VL22 to the vacuoles [18]. To understand how VSR1 recognizes the ctVSD of VL22, the PA domain (20–182) of VSR1 (VSR1‐PA) was cocrystallized with the C‐terminal pentapeptide (acetyl‐SDRFV) of VL22. After screening and optimization of crystallization conditions, diffraction‐quality crystals were obtained in 0.1 M MES pH 5.8, 30% (w/v) PEG 6000 and 0.1 M MES pH 7.0, 25% (w/v) PEG 6000, which were used to collect diffraction data to 1.90 and 1.95 Å, respectively (Table 1). Phases were solved by molecular replacement using the structure of the apo‐form of VSR1‐PA [16] as the search template. Residues Q51‐G53 and V179‐P182 of VSR1‐PA are disordered in the crystal structures. Apart from the fact that residue P50 is additionally disordered in the pH 7.0 structure, structures of VSR1‐PA determined at both pH are essentially identical, with a Cα r.m.s.d. value of 0.167 Å (Fig. 1A).

**Table 1:** Structure determination statistics.

Crystallization condition	0.1 M MES pH 5.8, 30% (w/v) PEG 6000	0.1 M MES pH 7.0, 25% (w/v) PEG 6000
PDB code	9L4O	9L4P
Data collection		
Space group	P 2_1_	P 2_1_
*a*, *b*, *c* (Å)	32.66, 60.38, 39.24	32.70, 60.44, 39.21
*α*, *β*, *γ* (°)	90, 113.38, 90	90, 113.29, 90
Resolution (Å)	36.02–1.90 (1.94–1.90)	36.01–1.95 (2–1.95)
No. of unique reflections	10 890 (648)	10 101 (713)
Multiplicity	3.7 (3.5)	2.9 (2.9)
Completeness	98.2 (92.9)	98.1 (99.0)
*R*_merge_ (%)	4.9 (30.1)	6.8 (58.6)
*I*/*σ*(*I*)	19.0 (4.4)	8.4 (1.7)
CC(1/2)	0.999 (0.885)	0.996 (0.788)
Refinement		
*R*_work_/*R*_free_	0.1687/0.2157	0.1744/0.2367
No. of atoms	1352	1296
Protein	1232	1201
Water	97	92
r.m.s. bond lengths (Å)	0.006	0.006
r.m.s. bond angles (°)	0.81	0.82
Ramachandran plot		
Favored/Allowed/Outliers (%)	97.5/2.5/0	98.1/1.9/0

### Binding of VL22 induces conformational changes in VSR1‐PA

**Fig. 2:**
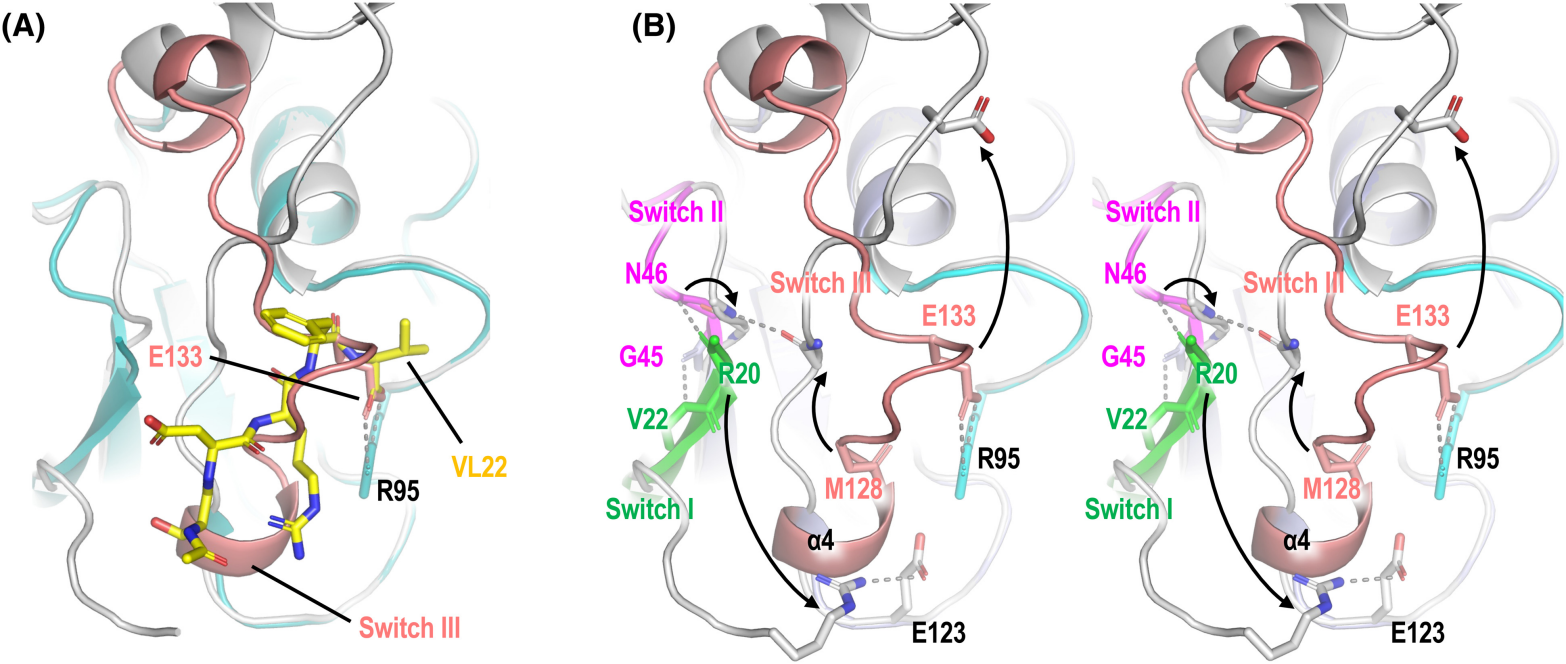
Conformational changes induced by binding of VL22. (A) Superimposition of apo (cyan) and VL22‐bound (gray) forms of VSR1‐PA. In the apo form, switch III residues (salmon) occupy the cargo‐binding site of VL22 (yellow). (B) A stereo view of the conformation changes (arrows) induced upon binding of VL22. The cargo‐binding loop, switch I, II, and III of the apo form of VSR1‐PA are shown in cyan, green, magenta, and salmon, respectively. The VL22/VSR1‐PA complex is in gray. Hydrogen bonds are indicated by dotted lines. Figures were generated by pymol. VL22, vicilin‐like seed storage protein 22; VSR1‐PA, vacuolar‐sorting receptor 1 protease‐associated domain.

The structure of VL22/VSR1‐PA complex was compared to that of the apo‐forms of the PA domain of VSR1 [16, 26]. Binding of VSR1‐PA to the C‐terminal pentapeptide sequence of VL22 induces a major conformational change in four regions that are denoted as switch I–IV (Fig. 1B and Fig. S1). The cargo‐binding site of VSR1‐PA is formed by residues in switch I, III, and a cargo‐binding loop consisting of a highly conserved _95_RGDCYF_100_ motif (Fig. 1B,C). In the apo‐form of VSR1‐PA, the cargo‐binding site is occupied by switch III residues (Fig. 2A). In particular, the carboxyl group of Glu133 occupies the position of the C‐terminal carboxyl group of VL22 and forms salt bridges to the invariant Arg95 of the RGDCYF motif (Fig. 2A). Binding of VL22 displaces switch III residues from the cargo‐binding site, causing α4 to unwind. Met128 of switch III and Asn46 of switch II move toward each other to form a backbone hydrogen bond (Fig. 2B). As a result, backbone hydrogen bonds between switch II (Asn46/Gly45) and switch I (Arg20/Val22) are broken, allowing Arg20 to move toward and form a salt bridge with Glu123 (Fig. 2B). Noteworthy, residues (Arg20, Val22, Gly45, Asn46, Glu123, Met128, Glu133) involved in conformational changes are highly conserved in VSR (Fig. 1C).

### Structural insight into how VSR1‐PA interacts with VL22

**Fig. 3:**
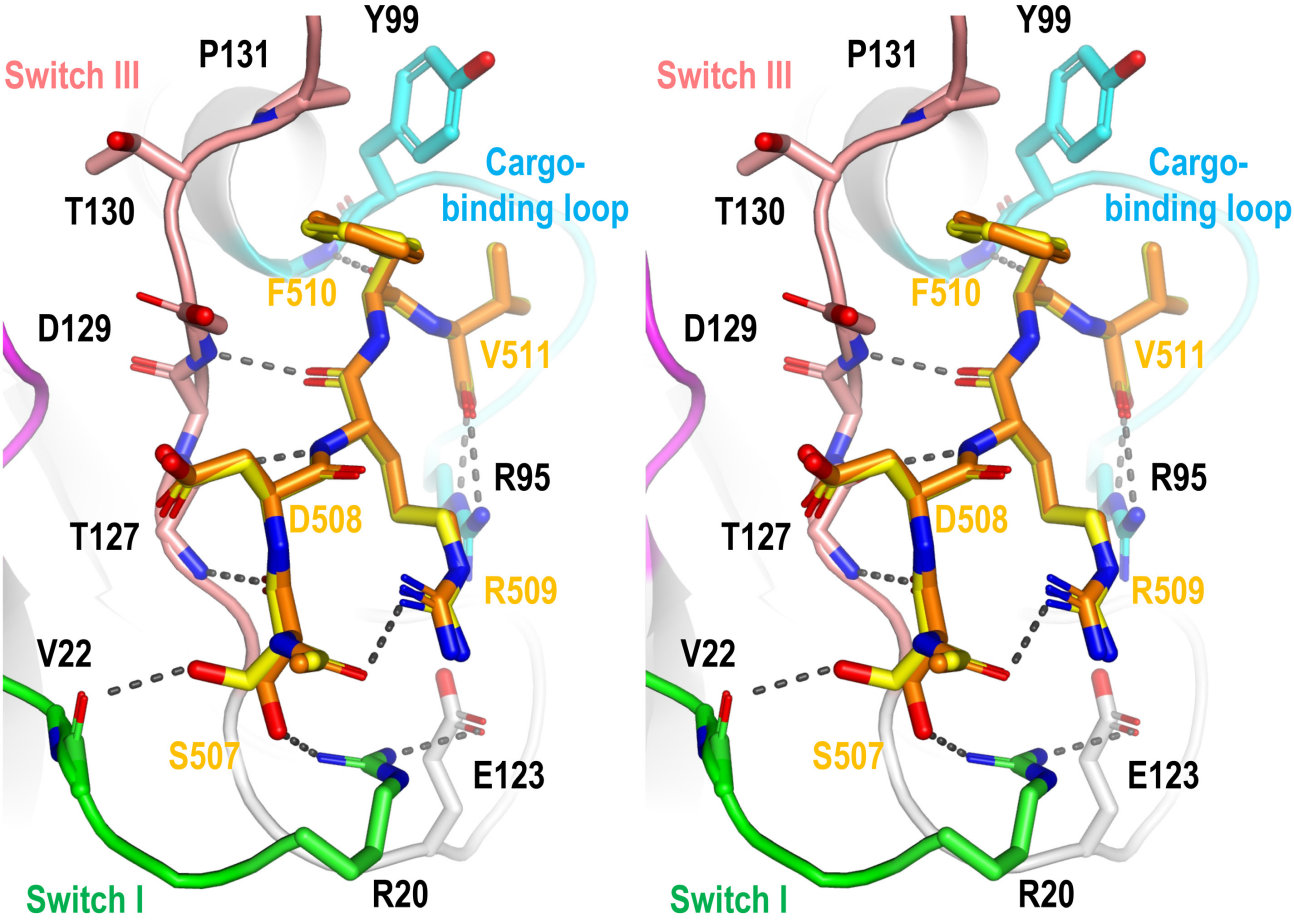
How VSR1 interacts with VL22. A stereo view of the cargo‐binding site of VL22/VSR1‐PA generated by pymol. Residues of switch I (green), switch III (salmon) and cargo‐binding loop (cyan) of VSR1 interact with the C‐terminal sequence of VL22. Structures of VL22 determined at pH 5.8 and 7.0 are represented in yellow and orange, respectively. Hydrogen bonds are indicated by dotted lines. VL22, vicilin‐like seed storage protein 22; VSR1‐PA, vacuolar‐sorting receptor 1 protease‐associated domain.

The conformation of the bound C‐terminal residues of VL22 (_507_SDRFV_511_) is well‐defined by electron densities in the crystal structures (Fig. S2). Unwinding of α4 upon cargo‐binding allows switch III residues Thr127‐Asp129 to form a short parallel β‐sheet with Ser507‐Arg509 of VL22 (Fig. 3). The C‐terminal carboxyl group of VL22 forms salt bridges with Arg95 of the cargo‐binding loop (Fig. 3). Phe510 of VL22 fits in a groove created by Tyr99, Asp129, Thr130, and Pro131 of VSR1. In particular, the conserved Tyr99 of the cargo‐binding loop forms hydrophobic interactions with both Phe510 and Val511 at the C terminus of VL22 (Fig. 3). The hydroxyl group of Ser507 of VL22 can form a hydrogen bond with the backbone carbonyl group of Val22 (in the pH 5.8 structure) or with the side chain of Arg20 (in the pH 7.0 structure) of switch I (Fig. 3). The side chain of Arg509 of VL22 folds back and forms a hydrogen bond with the N‐terminal acetyl group, which corresponds to the C=O group of Val506 of VL22 (Fig. 3). The conformation of switch I residues in the VL22/VSR1‐PA complex is stabilized by forming additional hydrogen bonds with Ser507 of VL22 and Glu123 of VSR1‐PA (Fig. 3).

### R509P and V511P substitutions abolished receptor‐cargo interaction and vacuolar sorting

**Fig. 4:**
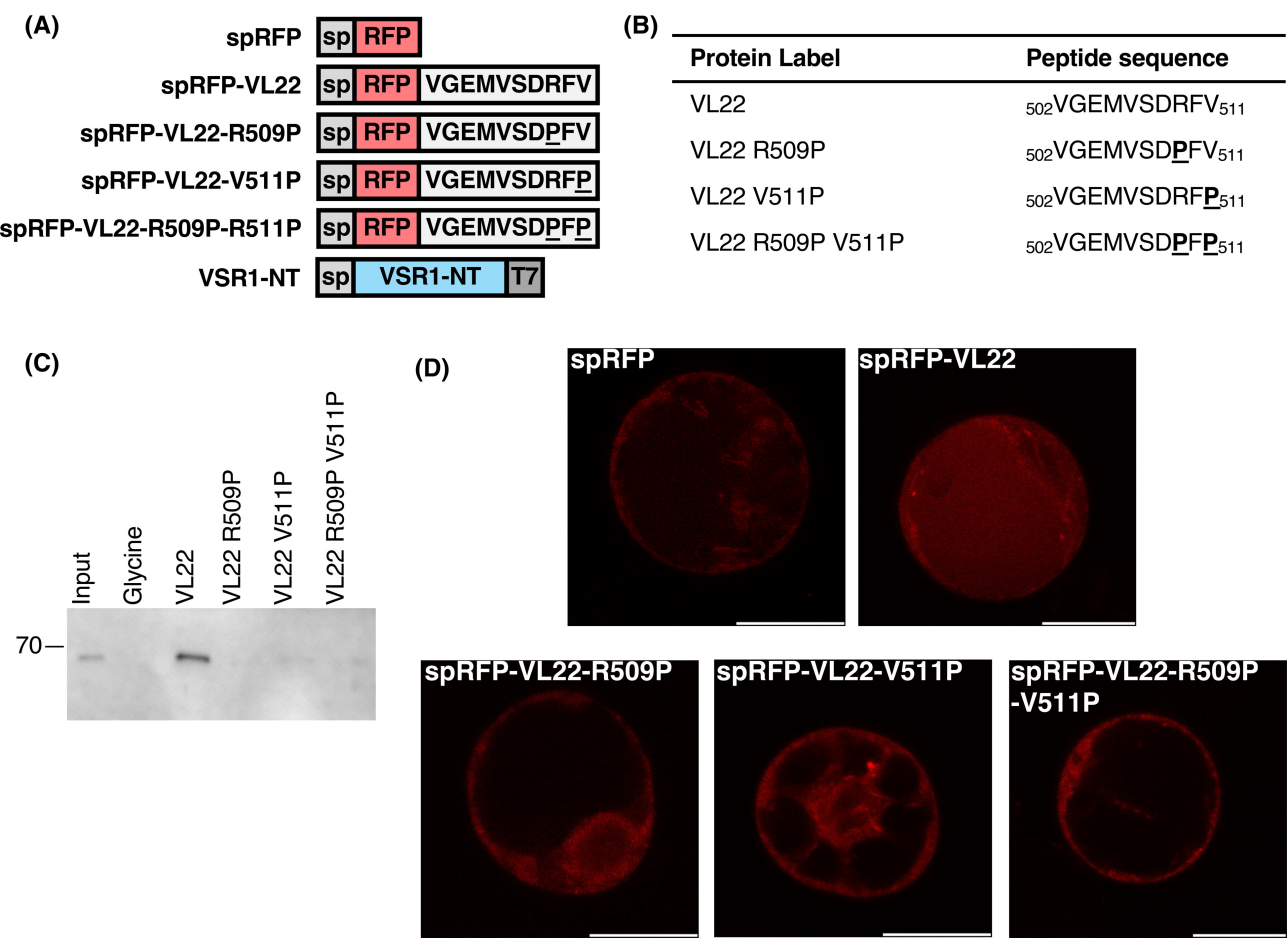
R509P and V511P substitutions in VL22 abolish receptor‐cargo interactions and vacuolar sorting. (A) Summary of constructs used. C‐terminal decapeptide sequences of vicilin‐like seed storage protein 22 (VL22) and its variants were tagged to the C‐terminus of secretory red fluorescent protein (spRFP) to create spRFP‐VL22, spRFP‐VL22‐R509P, spRFP‐VL22‐V511P, and spRFP‐VL22‐R509P‐V511P, respectively. sp: signal peptide. (B) Peptide sequences used in the pull‐down assay. Arg509 and Val511 of the C‐terminal decapeptide sequence of VL22 were replaced by proline (underlined). (C) Pull‐down assay. T7‐tagged vacuolar‐sorting receptor 1 N‐terminal domain (VSR1‐NT) was transiently expressed in *Arabidopsis* protoplasts and loaded to N‐hydroxysuccinimide activated resins coupled with glycine (negative control), the C‐terminal decapeptide sequences of VL22 or its variants listed in (B). After extensive washing to remove unbound proteins, samples were analyzed by immunoblotting using T7‐antibody (*n* = 2). Our results show that VSR1‐NT was co‐eluted with WT VL22 but not R509P, V511P, nor R509P‐V511P. (D) Confocal microscopy. spRFP, spRFP‐VL22, and its variants were transiently expressed in *Arabidopsis* protoplasts for 8 h before confocal imaging (scale bar = 20 μm) (*n* = 3). Tagging the spRFP with the C‐terminal sequence of VL22‐R509P, VL22‐V511P, and VL22‐R509P‐V511P did not sort the spRFP to the vacuoles.

Based on the crystal structures of VL22/VSR1‐PA, backbone φ angles of R509 and V511 of VL22 are −165° and −147°, respectively, which should disfavor the accommodation of proline with a φ angle ~ −60° due to the ring structure. Since proline lacks the backbone NH group, an R509P substitution should also break the hydrogen bond with the C=O group of Thr127 (Fig. 3). To test these structural insights, we introduced R509P and V511P substitutions to the C‐terminal sequence of VL22 and tested its interaction with VSR1 by pull‐down assay. T7‐tagged N‐terminal domain of VSR1 (VSR1‐NT) was loaded to NHS resins coupled with the C‐terminal decapeptide sequences of VL22 or its variants (VL22‐R509P, VL22‐V511P, VL22R509P‐V511P) (Fig. 4B). We showed that VSR1‐NT can interact with the C‐terminal sequence of VL22 but not VL22‐R509P, VL22‐V511P, nor VL22R509P‐V511P (Fig. 4C), suggesting that the proline substitutions abolished the interaction between VL22 and VSR1. To test the effect on vacuolar sorting, secretory red fluorescence protein (spRFP) tagged with the C‐terminal decapeptide sequence of VL22 or its variants was transiently expressed in *Arabidopsis* protoplasts (Fig. 4A,D). We showed that tagging VL22 to spRFP can redirect the protein to the vacuoles (Fig. 4D). On the other hand, spRFP tagged with VL22‐R509P, VL22‐V511P, and VL22‐R509P‐V511P mutants were not sorted to the vacuoles (Fig. 4D). These results suggest that R509P and V511P abolished the receptor–cargo interaction and the vacuolar sorting.

### C‐terminal sequence of VL43 cannot redirect spRFP to the vacuoles

**Fig. 5:**
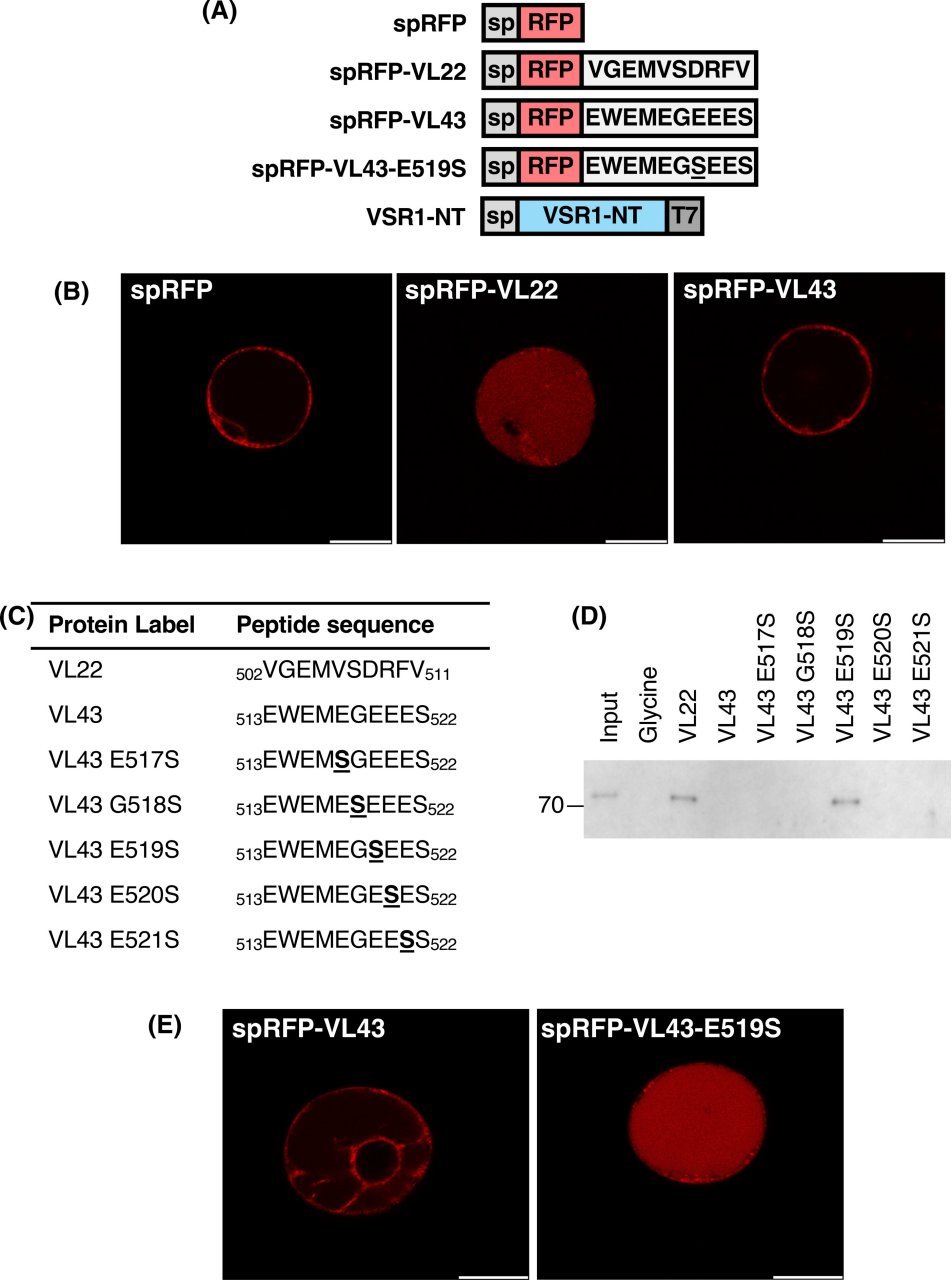
E519S substitution converts the C‐terminal sequence of VL43 to a ctVSD. (A) Summary of constructs used. C‐terminal decapeptide sequences of vicilin‐like seed storage protein 22 (VL22), vicilin‐like seed storage protein 43 (VL43) and its E519S variant were tagged to the C‐terminus of secretory red fluorescent protein (spRFP) to create spRFP‐VL22, spRFP‐VL43, and spRFP‐VL43‐E519S, respectively. sp: signal peptide. (B) Confocal microscopy. spRFP, spRFP‐VL22, and spRFP‐VL43 were transiently expressed in *Arabidopsis* protoplasts for 8 h before confocal imaging (scale bar = 20 μm) (*n* = 3). Tagging the spRFP with the C‐terminal sequence of VL43 did not sort the spRFP to the vacuoles. (C) Scanning mutagenesis. Residues of the C‐terminal decapeptide sequence of VL43 (E517‐E521) were systematically replaced by serine (underlined). (D) Pull‐down assay. T7‐tagged vacuolar‐sorting receptor 1 N‐terminal domain (VSR1‐NT) was transiently expressed in *Arabidopsis* protoplasts and loaded to NHS‐resins coupled with glycine (negative control), the C‐terminal decapeptide sequences of VL22, VL43, or its variants listed in (C). After extensive washing to remove unbound proteins, samples were analyzed by immunoblotting using T7‐antibody (*n* = 3). Our results show that VSR1‐NT was co‐eluted with the E519S variant of VL43. (E) Confocal microscopy. spRFP tagged with the C‐terminal sequence of VL43 or the E519S variant was transiently expressed in *Arabidopsis* protoplasts for 8 h before confocal imaging (scale bar = 20 μm) (*n* = 3). spRFP tagged with the E519S variant, but not the wild‐type sequence of VL43, was sorted to the vacuoles.

Compared to VL22, the C‐terminal sequence of VL43 (_513_EWEMEGEEES_522_) is rich in negatively charged glutamate residues (Fig. 5A). We have previously shown that the C‐terminal sequence of VL43 did not interact with VSR1 [18]. To test whether the C‐terminal sequence of VL43 can sort cargo to the vacuoles, we have tagged the C‐terminal sequences of VL22 and VL43 to the C terminus of spRFP (Fig. 5A) and transfected the constructs to *Arabidopsis* protoplasts (Fig. 5B). Our results show that tagging the C‐terminal sequence of VL22, but not VL43, can redirect spRFP to the vacuoles, suggesting that the C‐terminal sequence of VL43 is not a vacuolar sorting determinant.

### E519S substitution converts the C‐terminal sequence of VL43 to a ctVSD

We performed scanning mutagenesis on the C‐terminal sequence of VL43, where Glu517–Glu521 were systematically replaced by serine (Fig. 5C). To test the interaction between VSR1 and VL43 variants, we loaded T7‐tagged N‐terminal lumenal domain of VSR1 (VSR1‐NT) to NHS‐resins coupled with the C‐terminal decapeptide sequences of VL22, VL43, or its variants (Fig. 5D). Consistent with our previous findings, VSR1‐NT can interact with the C‐terminal sequence of VL22 but not VL43 (Fig. 5D). On the other hand, VSR1‐NT can bind to VL43‐E519S, suggesting that serine substitution on Glu519 of VL43 restores the interaction to VSR1. Next, we examined whether VL43‐E519S can redirect spRFP to the vacuoles. Secretory RFP tagged with the C‐terminal decapeptide sequence of VL43 and its E519S variant were transiently expressed in *Arabidopsis* protoplasts (Fig. 5E). The spRFP tagged with the VL43‐E519S mutant, but not the wild‐type VL43 sequence, was observed in the vacuoles. This result suggests that a single substitution of E519S converts the sequence of VL43 to a ctVSD that can sort cargo proteins to the vacuoles (Fig. 5E).

## Discussion

The PA domain of VSR is responsible for recognizing the C‐terminal vacuolar sorting determinant (ctVSD) on the cargo proteins and sorting them to the vacuoles. We have determined the structure of VSR1‐PA in complex with the ctVSD of cruciferin 1 [18] and, in this study, the structure of VSR1‐PA in complex with the ctVSD of VL22 (Fig. 1). A common theme emerges—the C‐terminal carboxyl group of the ctVSD is recognized by forming salt‐bridges with the invariant arginine, Arg95, of the cargo‐binding loop, and the ctVSD forms several backbone hydrogen bonds with the cargo‐binding loop and switch III residues (Fig. 3). As a result, the proline residue that could break the backbone hydrogen bonds is not preferred for ctVSD‐VSR interaction. For example, a proline residue at the −3 position of the ctVSD (Pro453 in the case of cruciferin 2) prevents VSR binding, and a P453A substitution converts the C‐terminal sequence of cruciferin 2 to a ctVSD sorted by VSR [18]. Moreover, proline is not allowed at the −1 position due to the restriction of the backbone φ angle by the ring structure. In this study, we further showed that proline substitutions at −3 and −1 positions (Arg509 and Val511 in the case of VL22) abolished the receptor–cargo interactions and vacuolar sorting (Fig. 4). Taken together, proline is not allowed at −3 and −1 positions of ctVSD recognized by VSR.

**Fig. 6:**
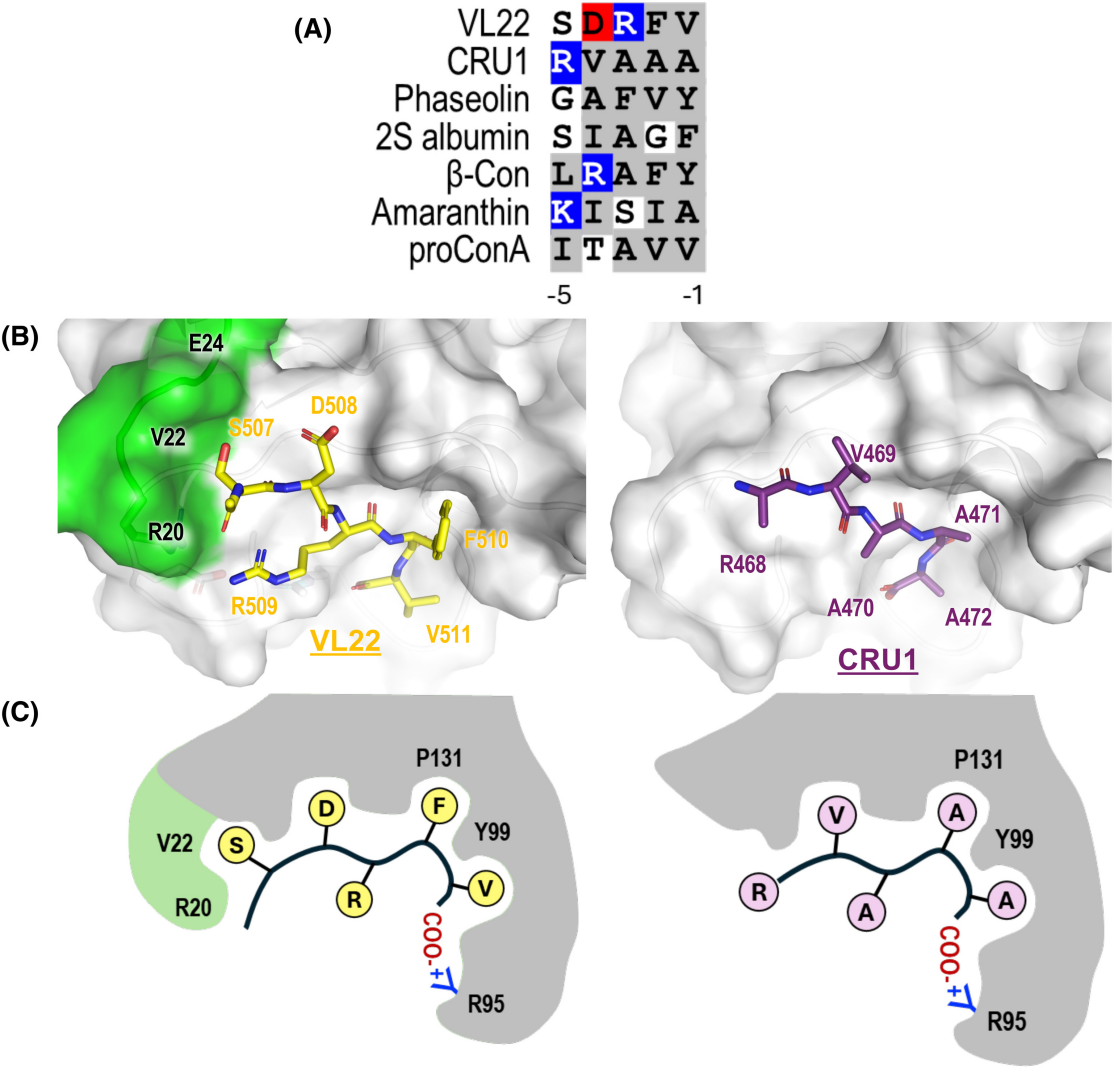
A summary of how VSR recognizes ctVSD. (A) C‐terminal vacuolar sorting determinant (ctVSD) of seed storage proteins is rich in hydrophobic residues. The last 5 residues of vicilin‐like seed storage protein 22 (VL22) (Uniprot ID; Q9SK09) and cruciferin 1 (CRU1) (P15455) from *Arabidopsis thaliana*, phaseolin from common bean (P07219), 2S albumin from Brazil nut (P04403), β‐conglycinin (β‐Con) from soybean (P11827), amaranthin from Prince's Feather (Q38712), and pro‐Concanavalin A (proConA) from jack bean (P02866) are shown. Hydrophobic, acidic, and basic residues are shaded in gray, red, and blue, respectively. The residue at the −1 position refers to the residue at the C‐terminus. (B) Two binding modes of ctVSD‐VSR interactions. In the VL22/VSR1‐PA structure (left), switch I residues R20‐E24 (green) of VSR1 are structured and form part of the binding site for VL22. In contrast, these residues are disordered in the CRU1/VSR1‐PA structure (right). Figures were generated by pymol. (C) A schematic diagram showing switch I residues (green) create an extra binding pocket for the −5 residue in VL22/VSR1‐PA. VSR, vacuolar‐sorting receptor; VSR1‐PA, vacuolar‐sorting receptor 1 protease‐associated domain.

The structure of VL22/VSR1‐PA reported here illustrates another binding mode of ctVSD‐VSR. Compared to the structure of VSR1‐PA in complex with cruciferin 1 (CRU1), switch I residues Arg20‐Glu24 become structured and form part of the binding site for VL22 (Fig. 6B,C). The switch I residues are stabilized by hydrogen bonds with Ser507 of VL22 and Glu123 of VSR1 (Fig. 3). These additional conformational changes are facilitated by having a small serine residue at the position −5 of the ctVSD (Ser507 in the case of VL22). The corresponding residue in CRU1 is a bulkier residue, Arg468, which prevents switch I residues from approaching the cargo‐binding site due to steric hindrance. As a result, switch I residues (Arg20‐Glu24) and the side chain of Arg468 are disordered in the CRU1/VSR1‐PA structure (Fig. 6B,C). The binding mode of VL22 provides additional interactions with VSR, which could explain why the ctVSD of VL22 has a stronger binding affinity to VSR1‐PA compared to CRU1 [18].

No consensus sequence has been identified for ctVSD, except that the C‐terminal sequences of seed storage proteins are rich in hydrophobic residues. The C‐terminal sequences of known ctVSD that can interact with VSR [18, 29, 38, 39, 40, 41, 42] are shown in Fig. 6A. We have modeled the structures of these ctVSD in complex with VSR1‐PA (Fig. S3). For ctVSD sequences with a small residue at the −5 position (i.e., phaseolin and 2S albumin), the structures could be modeled based on the crystal structure of VL22/VSR1‐PA (Fig. S3E,F). However, ctVSD sequences containing a large residue at the −5 position (i.e., β‐conglycinin, amaranthin, and pro‐Concanavalin A) could not adopt the VL22‐binding mode due to steric clashes with Arg20‐Glu24 of switch I. They are modeled based on the crystal structure of CRU1/VSR1‐PA (Fig. S3G–I). In all cases, the hydrophobic residues at the −1 and −2 positions of ctVSD are recognized by forming hydrophobic interactions with Tyr99 and Pro131 of VSR (Figs 6 and S3). Tyr99 and Pro131 are highly conserved in VSR in the sequence alignment (Fig. 1C), justifying the observation that residues at the −1 and −2 positions are predominantly hydrophobic (Fig. 6A). In addition, the arginine residue at the −4 position of β‐conglycinin (Fig. S3G) and the lysine residue at the −5 position of amaranthin (Fig. S3H) could form favorable electrostatic interactions with Asp129 and Glu123, respectively, of VSR1.

**Fig. 7:**
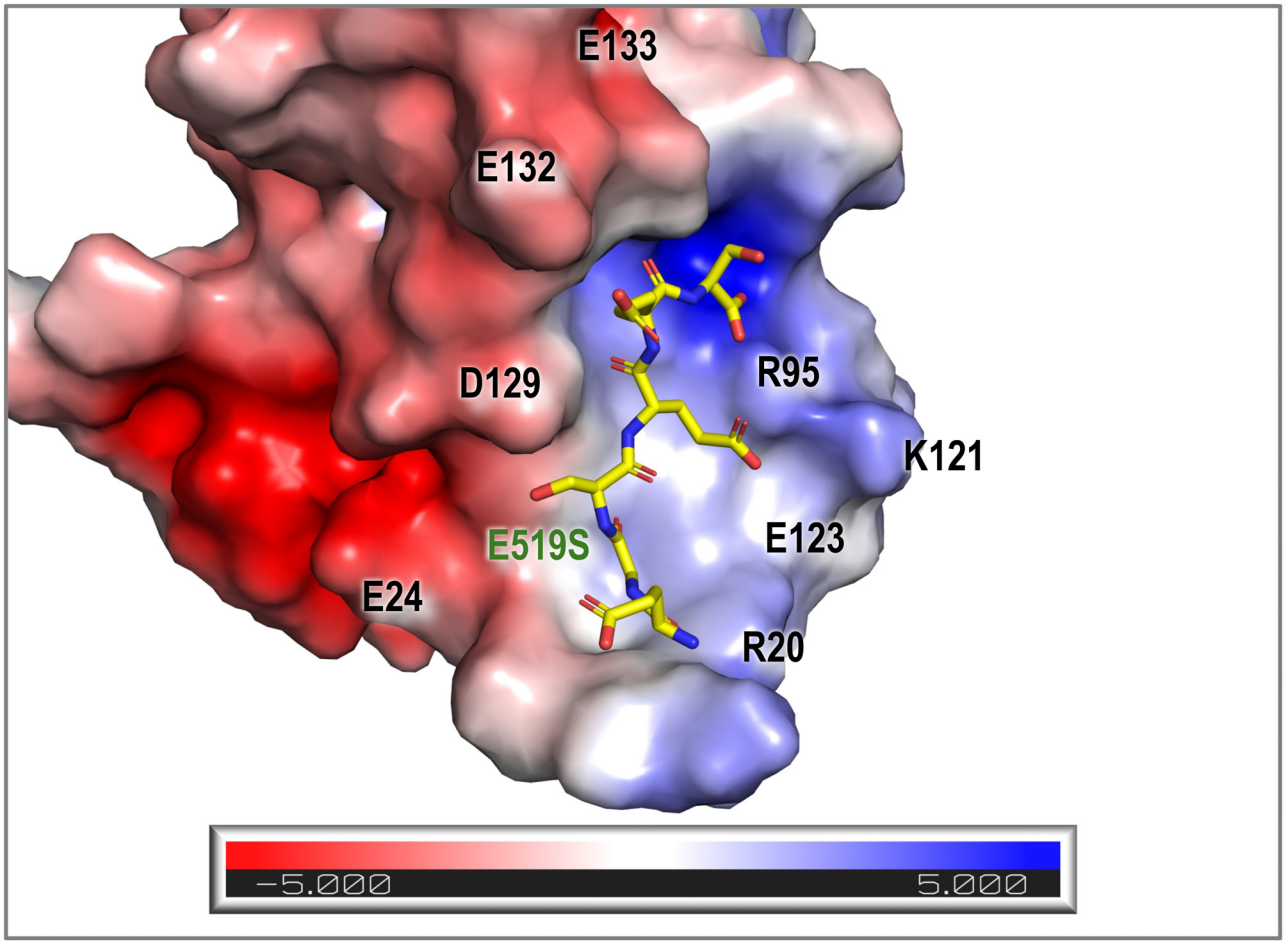
A model of VL43‐E519S in complex with VSR1‐PA. The structure of VL43‐E519S (yellow) was modeled in molecular graphics application software coot and minimized in molecular dynamics package GROMACS. Electrostatic potential of VSR1‐PA calculated by Advanced Poisson‐Boltzmann Solver implemented in pymol is represented by a red‐to‐blue gradient. The E519S substitution avoids a charge–charge repulsion with the negative electrostatic potential near Glu24 and Asp129. VL43, vicilin‐like seed storage protein 43; VSR1‐PA, vacuolar‐sorting receptor 1 protease‐associated domain.

We also demonstrate that electrostatic repulsion is unfavorable for ctVSD‐VSR binding. VSR1 did not interact with the C‐terminal sequence of VL43 and, thus, did not sort spRFP tagged with VL43 to the vacuoles (Fig. 5B). As VL43 contains many negatively charged glutamate residues at its C terminus, we argued that charge–charge repulsion prevents VL43 from interacting with VSR1. To address this hypothesis, we performed scanning mutagenesis and found that the E519S substitution converts the C‐terminal sequence of VL43 to a vacuolar sorting determinant that can interact with VSR1 (Fig. 5D,E). We have modeled the structure VSR1‐PA in complex with VL43‐E519S and calculated the electrostatic surface of VSR1‐PA (Fig. 7). The electrostatic potential of the cargo‐binding site is asymmetric: acidic residues Glu24, Asp129, Glu132, and Glu133 create a strong negative potential on one side of VSR1‐PA, while Arg20, Arg95, and Lys121 create a positive potential on the other side (Fig. 7). The model also suggests that Glu519 of VL43, which points to a strong negative potential between Glu24 and Asp129 of VSR1, should create the strongest charge–charge repulsion and justify the observation that the E519S substitution can convert the C‐terminal sequence of VL43 to a ctVSD.

Our results suggest that the PA domain of VSR interacts with up to five residues of ctVSD (Fig. 6), which is consistent with the observation that the shortest C‐terminal sequences necessary and sufficient for vacuolar sorting are in the range of 4–5 residues [43]. For example, tagging the AFVY motif of phaseolin [44] and the KISIA motif of amaranthin [40] to fluorescent proteins is sufficient to redirect the reporter proteins to the vacuoles. In other words, the sequence specificity for vacuolar sorting should mainly be determined by the interactions between the PA domain and the ctVSD.

The recognition of ctVSD by VSR is likely promiscuous; that is a result of the additive effect of individual side chains of ctVSD interacting with the PA domain of VSR. In summary, Arg95 of the PA domain recognizes the C‐terminal carboxyl group and anchors the ctVSD residues at positions −1 to −5 to the cargo‐binding sites (Fig. 6C). Residues at the −1 and −2 positions prefer hydrophobic residues because they can form favorable interactions with Tyr99 and Pro131 of the PA domain. Residue at the −3 position should favor an arginine (Arg509 in the case of VL22) because it can fold back to form a hydrogen bond to the backbone amide of the ctVSD and a long‐range electrostatic interaction with Glu123 (Fig. 3). Residue at the −4 position should favor basic residue over acidic one because it faces the strongest negative electrostatic potential of the PA domain near Glu24 and Asp129 (Fig. 7). A serine residue at the −5 position should promote the VL22 binding mode that allows extra interaction between ctVSD and switch I residues (Arg20 and Val22). On the other hand, proline residues that disrupt backbone hydrogen bonds or backbone conformation are not allowed at the −3 and −1 positions (Fig. 4). The recognition of ctVSD is likely an additive effect, as the ctVSD of VL22 has residues providing favorable interactions at −1, −2, −3, and −5 positions, but an unfavorable acidic residue at the −4 position. Such promiscuous recognition also explains why no consensus sequence was found in the ctVSD except that the last two residues are predominantly hydrophobic (Fig. 6). As residues (cargo‐binding loop, switch I and III) involved in binding ctVSD are highly conserved (Fig. 1C), the insights provided in our study could be generalized to how seed storage proteins are sorted to the vacuoles in other species.

## Materials and methods

### Crystallization and structure determination

Expression and purification of VSR1‐PA (residues 20–182) were described previously [16, 18]. The purified VSR1‐PA was concentrated to 15 mg·mL^−1^ and cocrystallized with a 5‐fold molar excess of the C‐terminal pentapeptide of VL22 (acetyl‐SDRFV). Initial crystallization conditions were obtained by screening with commercial kits (Crystal Screen 1 & 2, Index 1 & 2 [Hampton Research, Aliso Viejo, CA, USA], JCSG Core 1–4 and JCSG ProComplex [Qiagen, Venlo, The Netherlands]) using the sitting‐drop‐vapor‐diffusion method. After optimization of pH and precipitant concentration, the protein crystals for diffraction data collection were obtained in 0.1 M MES, pH 5.8, 30% (w/v) PEG 6000 or 0.1 M MES, pH 7.0, 25% (w/v) PEG 6000. Diffraction data were collected using the in‐house X‐ray generator (FR‐X; Rigaku, Tokyo, Japan) equipped with a hybrid direct photon detector (EIGER 4 M; Destris AG, Baden, Switzerland), processed with XDS, and phased by molecular replacement using the VSR1‐PA structure (PDB: 4TJV) as the search model [45, 46]. Models were built interactively using coot and refined using PHENIX.REFINE [47, 48].

### Pull‐down assay

Synthetic decapeptides of the C‐terminal sequences of VL22 (VGEMVSDRFV), VL22‐R509P (VGEMVSDPFV), VL22‐V511P (VGEMVSDRFP), VL22‐R509P‐V511P (VGEMVSDPFP), VL43 (EWEMEGEEES), VL43‐E517S (EWEMSGEEES), VL43‐G518S (EWEMESEEES), VL43‐E519S (EWEMEGSEES), VL43‐E520S (EWEMEGESES), VL43‐E521S (EWEMEGEESS) were coupled to NHS‐activated Sepharose™ 4 Fast Flow resins (GE Healthcare, Chicago, IL, USA) following the manufacturer's instructions. After T7‐tagged VSR1‐NT was transiently expressed in *Arabidopsis* PSBD protoplast [49], the culture medium was dialyzed against the VSR binding buffer (25 mm HEPES, 150 mm NaCl, 1 mm MgCl_2_, 1 mm CaCl_2_, pH 7.1) supplemented with protease inhibitor cocktail (Roche cOmplete™, EDTA‐free Protease Inhibitor Tablets; Sigma‐Aldrich, St. Louis, USA) at 4 °C. Dialyzed culture medium was 0.22 μm‐filtered and incubated at 4 °C overnight with peptide‐coupled NHS‐resins pre‐equilibrated with the VSR binding buffer supplemented with 0.35% CHAPS. The NHS‐resins were washed with the VSR binding buffer supplemented with 1% CHAPS extensively. Bound proteins were analyzed by SDS/PAGE and immunoblot using T7 antibody (Abcam, Cambridge, UK).

### Plasmid construction for functional study in *Arabidopsis* protoplasts

All fluorescent fusion constructs used in this study were cloned into a premade spRFP backbone in plant expression vector pBI221. ctVSD decapeptide sequences were inserted at the C‐terminus of spRFP with homologous recombination using the ClonExpress II One Step Cloning kit (Vazyme Biotech, Nanjing, China) following the manufacturer's instructions. Sequences of all constructs were verified by DNA sequencing.

### Transient expression in *Arabidopsis* PSBD protoplasts and confocal imaging

Transient expression of proteins with fluorescent tags and culture methods of *Arabidopsis* PSBD protoplasts were performed as described previously [18]. Confocal images were acquired 8 h after transformation using the Leica SP8 system. Cells were observed under a 63 × NA1.20 (water) objective, and RFP/mCherry signals were excited by 552 nm. Images were captured and processed with the Leica software (Leica Application Suite X 3.7.2.22383).

### Modeling of VSR1‐PA in complex with the C‐terminal sequences of seed storage proteins

The C‐terminal sequences of phaseolin and 2S albumin were modeled based on the crystal structure of VL22/VSR1‐PA, and those of β‐Con, proConA, and amaranthin were modeled based on the crystal structures of CRU1/VSR1‐PA. The side chain rotamers of the cargo sequences were selected interactively using the program coot [47] to avoid steric clashes, followed by energy minimization using the program chimera [50].

### Modeling of VL43‐E519S in complex with VSR1‐PA

The model was created in coot [47] based on the crystal structures of VL22/VSR1‐PA and minimized in GROMACS 2024 using the AMBER99sb‐ILDN force field [51, 52]. Calculation of electrostatic potential was carried out by Adaptive Poisson‐Boltzmann Solver (APBS) implemented in the program pymol [53].

## Conflict of interest

The authors declare no conflict of interest.

## Author contributions

SNL, H‐ET, AH‐FL, LJ, and KBW designed experiments; SNL and H‐ET performed experiments; SNL and K‐BW analyzed data and wrote the manuscript; LJ and K‐BW acquired funding and provided essential resources; K‐BW conceived and supervised the study.

## Supporting information


**Fig. S1.** Binding of ctVSD of VL22 induces conformational changes in 4 regions of VSR1‐PA.
**Fig. S2.** Bound VL22 is well‐defined in the crystal structure.
**Fig. S3.** Two binding modes of seed storage proteins ctVSDs/VSR1‐PA.

## Data Availability

Accession codes: The crystal structures of *A. thaliana* VSR1‐PA in complex with the C‐terminal pentapeptide of VL22 at pH 5.8 and pH 7.0 have been deposited in the Protein Data Bank (PDB codes: 9L4O and 9L4P). *Arabidopsis* sequences used in this study can be accessed in The Arabidopsis Information Resource database with the following accession numbers: VSR1, At3g52850; CRU1, At5g44120; VL22, At2g28490; VL43, At4g36700.
