# Supplementary material for: Structural insights into how vacuolar sorting receptor recognizes the C‐terminal sorting determinant of a vicilin‐like seed storage protein
Source: FEBS J. 2025 Sep 2;293(1):257–70. doi: 10.1111/febs.70245 (PMC12796998; doi:10.1111/febs.70245)
Supplement: Supplementary file 1 — Fig. S1. Binding of ctVSD of VL22 induces conformational changes in 4 regions of VSR1‐PA. Fig. S2. Bound VL22 is well‐defined in the crystal structure. Fig. S3. Two binding modes of seed storage proteins ctVSDs/VSR1‐PA. [file FEBS-293-257-s001.pdf]

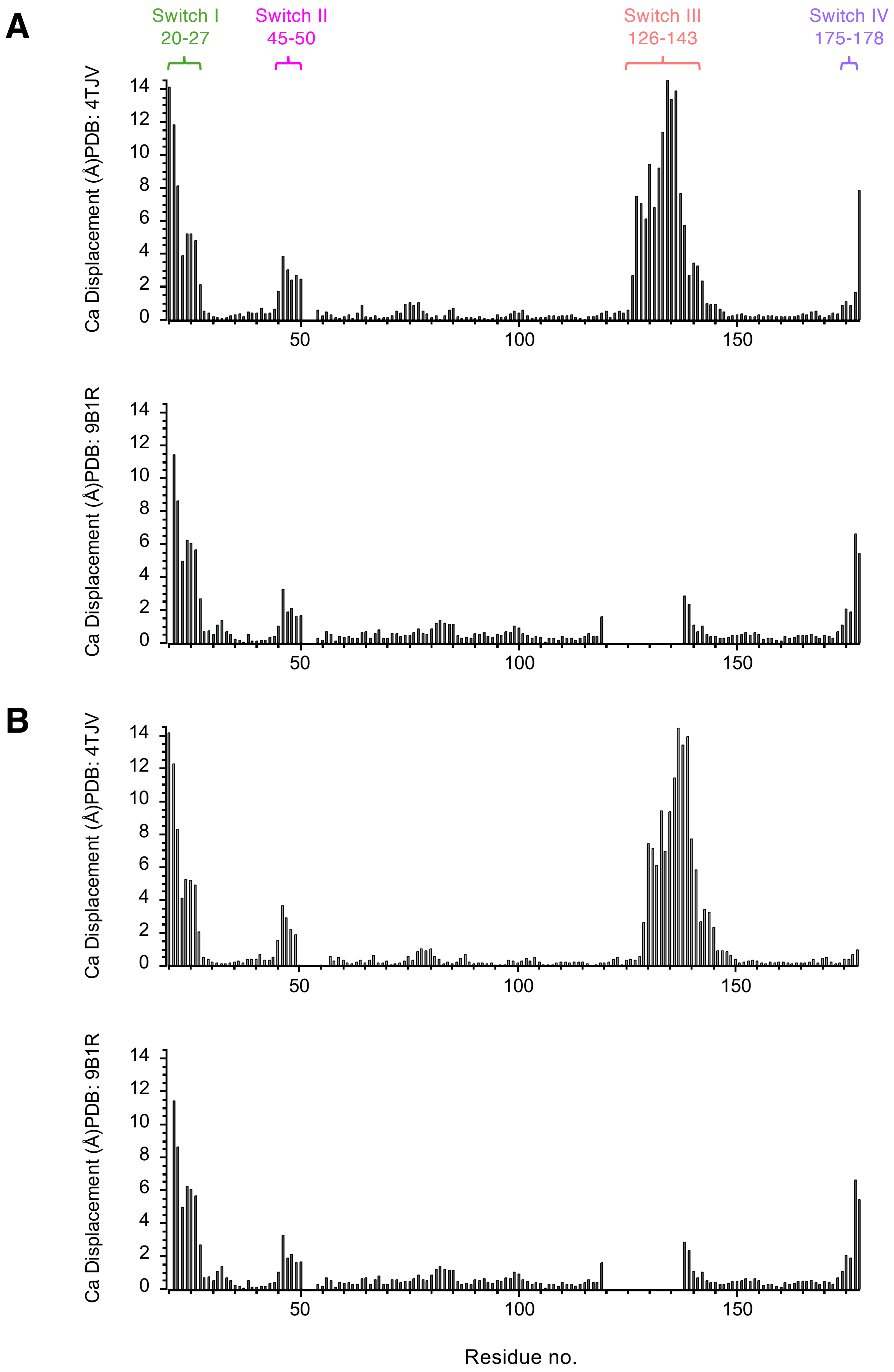

Figure S1. Binding of ctVSD of VL22 induces conformational changes in 4 regions of VSR1-PA. Structures of VL22/VSR1-PA determined at (A) pH 5.8 and (B) pH 7.0 were superimposed to the crystal structures of the apo-form of VSR1-PA (PDB: 4TJV) and VSR1-NT (PDB: 9B1R) and values of Cα displacement were calculated using the program CHIMERA. Large values of Cα displacement were found in four distinct regions of VSR1-PA: switch I (20-27), switch II (45-50), switch III (126-143) and switch IV (175-178).

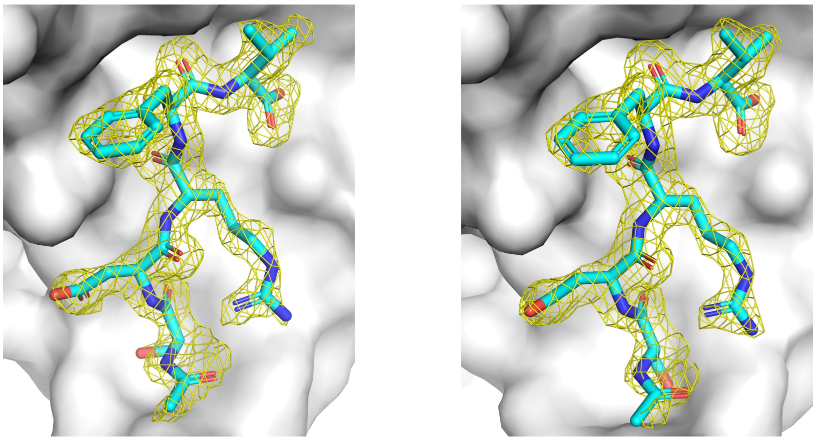

pH5.8 **pH7.0**

Figure S2. Bound VL22 is well defined in the crystal structure. Electron densities (2fo-fc, yellow) of the C-terminal pentapeptide (acetyl-SDRFV, cyan) of VL22 are contoured at 1σ. All residues including the N-terminal acetyl group are well defined in the crystal structures of VL22/VSR1-PA complex determined at pH 5.8 and 7.0. Surfaces of the cargo-binding site of VSR1-PA are shown in the background. Figures were generated by PyMOL.

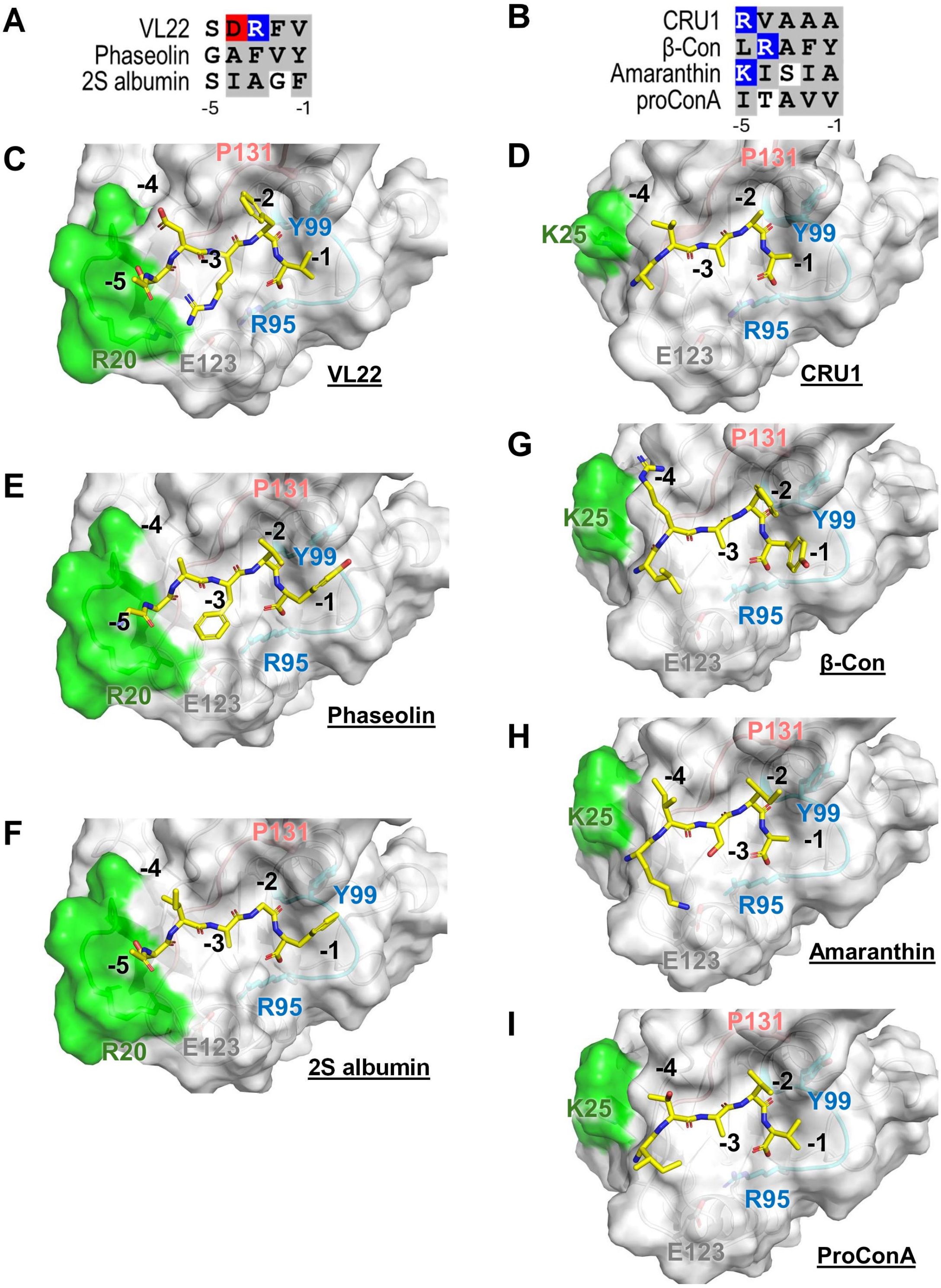

Figure S3. Two binding modes of seed storage proteins ctVSDs/VSR1-PA.
(A) With a small residue at the -5 position, the binding mode of phaseolin and 2S albumin should be similar to that of VL22/VSR1-PA. (B) With a bulkier residue at the -5 position, the binding mode of β-conglycinin (β-Con), amaranthin and pro-Concanavalin A (proConA) should be similar to that of CRU1/VSR1-PA. (C) In the crystal structure of VL22/VSR1-PA, switch I residues R20-E24 (green) of VSR1-PA are structured and form extra interactions with a serine residue at the -5 position of VL22. (D) In the crystal structure of CRU1/VSR1-PA, these switch I residues are unstructured due to steric clashes with a bulkier arginine residue at the -5 position. (E-I) The C-terminal sequences of phaseolin and 2S albumin were modelled based on the crystal structure of VL22/VSR1-PA, and those of β-Con, amaranthin and proConA were modeled based on the crystal structures of CRU1/VSR1-PA. The side chain rotamers of the ctVSD sequences were selected interactively using the program COOT to avoid steric clashes, followed by energy minimization using the program CHIMERA. VSR1-PA is shown in surface representation. Cargo-binding loop, switch I, switch III, and ctVSD are in cyan, green, salmon and yellow, respectively. Figures were generated by PyMOL.
